# Implementation of a unilateral hip flexion exosuit to aid paretic limb advancement during inpatient gait retraining for individuals post-stroke: a feasibility study

**DOI:** 10.1186/s12984-024-01410-0

**Published:** 2024-07-18

**Authors:** Chih-Kang Chang, Christina Lee, Richard W. Nuckols, Asa Eckert-Erdheim, Dorothy Orzel, Maxwell Herman, Jennifer Traines, Sara Prokup, Arun Jayaraman, Conor J. Walsh

**Affiliations:** 1 John A. Paulson School of Engineering and Applied Sciences, Harvard University, Boston, MA USA; 2 Mechanical and Industrial Engineering, University of Massachusetts Lowell, Lowell, MA USA; 3 Shirley Ryan AbilityLab, Chicago, IL USA; 4 Feinberg School of Medicine, Northwestern University, Chicago, IL USA

**Keywords:** Soft exosuit, Assistive device, Stroke rehabilitation, Physical therapy, Inpatient rehabilitation

## Abstract

**Background:**

During inpatient rehabilitation, physical therapists (PTs) often need to manually advance patients’ limbs, adding physical burden to PTs and impacting gait retraining quality. Different electromechanical devices alleviate this burden by assisting a patient’s limb advancement and supporting their body weight. However, they are less ideal for neuromuscular engagement when patients no longer need body weight support but continue to require assistance with limb advancement as they recover. The objective of this study was to determine the feasibility of using a hip flexion exosuit to aid paretic limb advancement during inpatient rehabilitation post-stroke.

**Methods:**

Fourteen individuals post-stroke received three to seven 1-hour walking sessions with the exosuit over one to two weeks in addition to standard care of inpatient rehabilitation. The exosuit assistance was either triggered by PTs or based on gait events detected by body-worn sensors. We evaluated clinical (distance, speed) and spatiotemporal (cadence, stride length, swing time symmetry) gait measures with and without exosuit assistance during 2-minute and 10-meter walk tests. Sessions were grouped by the assistance required from the PTs (limb advancement and balance support, balance support only, or none) without exosuit assistance.

**Results:**

PTs successfully operated the exosuit in 97% of sessions, of which 70% assistance timing was PT-triggered to accommodate atypical gait. Exosuit assistance eliminated the need for manual limb advancement from PTs. In sessions with participants requiring limb advancement and balance support, the average distance and cadence during 2-minute walk test increased with exosuit assistance by 2.2 ± 3.1 m and 3.4 ± 1.9 steps/min, respectively (*p* < 0.017). In sessions with participants requiring balance support only, the average speed during 10-meter walk test increased with exosuit by 0.07 ± 0.12 m/s (*p* = 0.042). Clinical and spatiotemporal measures of independent ambulators were similar with and without exosuit (*p* > 0.339).

**Conclusions:**

We incorporated a unilateral hip flexion exosuit into inpatient stroke rehabilitation in individuals with varying levels of impairments. The exosuit assistance removed the burden of manual limb advancement from the PTs and resulted in improved gait measures in some conditions. Future work will understand how to optimize controller and assistance profiles for this population.

**Supplementary Information:**

The online version contains supplementary material available at 10.1186/s12984-024-01410-0.

## Background

Stroke is a leading cause of adult disability in the United States, affecting nearly 800,000 individuals annually [1]. Functional impairment of individuals post-stroke increases the risk of falls and reduces quality of life [1]. Post-stroke physical therapy focuses on recovering the ability to walk and improving walking quality [2, 3]. Physical therapy involving repetitive mass practice and task-specific training has shown positive results in motor recovery [4–6], with the amount of practice during training positively associated with gait relearning [7, 8]. In addition, individuals experience rapid changes in their neuromotor pathway within three months following stroke incident, often defined as subacute phase of stroke [9]. Unsurprisingly, inpatient rehabilitation during this period has a large impact on the motor recovery, expected mobility, and independence in activities of daily living, especially for severely to moderately affected patients [10].

However, the significant mobility deficits exhibited by individuals with subacute stroke adds substantial physical burdens on physical therapists (PTs) during inpatient rehabilitation. Specifically, PTs may experience difficulty with manual lifting, static holding, and maintaining challenging postures which are necessary therapeutic handling [11, 12]. To promote safe ambulation for individuals with low mobility during gait retraining therapies, PTs must not only support body weight and assist with balance control, but also advance the patients’ limbs manually [13, 14] mainly due to their weakened hip flexors at movement initiation [15]. Coordinating these activities leads to high loads on the PT’s musculoskeletal system and induces a high risk of work-related disorders [11, 16]. Therefore, reliance on manual assistance provided by PTs makes it challenging for patients to receive mass practice that is essential in promoting motor recovery during inpatient rehabilitation [3, 17, 18].

In the past few decades, various electromechanical devices have been developed to assist ambulation of patients and reduce musculoskeletal load in PTs by providing body weight support and limb advancement assistance during gait retraining [16]. Examples of these devices include partial body weight supported treadmill training (PBWSTT) [19], end-effector-type gait devices [20], and portable exoskeletons [21, 22]. The use of electromechanical devices allows severely impaired patients to receive repetitive mass practice early [23, 24] and reduces the physical burden experienced by PTs. Interventions incorporating these devices have been shown to provide either improved or similar benefits compared to conventional therapy [13, 19, 22, 24–35]. However, while there has been recent development of electromechanical devices that enable gait retraining in diverse environments [36], the majority are limited to being used overground or on a treadmill [19–22]. Moreover, existing devices can involve substantial setup time, a high learning curve [37–39], or multiple PTs to operate [19, 27, 40]. Over the progression of rehabilitation, individuals post-stroke may no longer require substantial body weight support [26, 41]; however, they may continue to require limb advancement assistance across all stages of recovery [15, 42]. Devices that can assist limb advancement in a diverse range of activities and environments have the potential to promote gait relearning during inpatient rehabilitation more effectively.

The use of soft exosuits has been explored as a mean to deliver assistance for individuals post-stroke [43–50]. Contrary to rigid exoskeletons, exosuits do not provide body weight support. Instead, they utilize a lightweight and non-restrictive approach to allow assistance to supplement an individual’s walking capacity. Previous studies have evaluated the effect of exosuit assistance in individuals with chronic stroke. In these studies, exosuits effectively targeted the ankle to provide both immediate (i.e., orthotic effect) [43] and rehabilitative (i.e., therapeutic effect) [44–46] functional and biomechanical benefits in individuals post-stroke. Recently, a few preliminary studies have demonstrated a positive orthotic effect in biomechanical strategies of exosuits targeting the hip in individuals with chronic stroke [47, 48, 50]. The weakened hip flexors typical in individuals in their early stages of stroke recovery often contribute to difficulty in limb advancement and require manual assistance from PTs. Therefore, we anticipate that exosuit providing hip flexion assistance may be particularly useful during inpatient rehabilitation for individuals post-stroke to reduce the physical burden of PTs, promote mass practice, and maximize recovery progress. Unfortunately, the use of hip exosuits to aid inpatient rehabilitation during the early stages of stroke recovery is unexplored.

The objective of this study was to assess the feasibility of implementing a unilateral hip flexion exosuit as a tool to aid paretic limb advancement during inpatient gait retraining for individuals post-stroke. We describe the design of the exosuit, focusing on the integration of different suit components that enabled PTs to use the exosuit without in-person technical support. The exosuit was used concurrently with standard inpatient rehabilitation for 14 patients, each for one to two weeks. We quantified various clinical and spatiotemporal measures to investigate the role of our exosuit in reducing PT burden and improving the walking capacity of individuals post-stroke with various levels of impairment over the course of their recovery.

## Methods

### Exosuit textile and hardware design

We updated a hip flexion exosuit previously developed in Harvard Biodesign Lab [50, 51] to enable the operation of the exosuit by the PTs without in-person technical support. Specifically, the exosuit was modified such that all components could be assembled as a single unit and be easily donned within minimal time (1 min 35 s; see additional files 1 and 2). The adjustable straps connecting the thigh wraps and the waist belt allowed seamless integration of different sensors without tangling and prevented them from falling. Although the exosuit provided assistance unilaterally, the suit consisted of bilateral thigh wraps to accommodate different paretic sides of patients with minimal modification on the device.

**Fig. 1:**
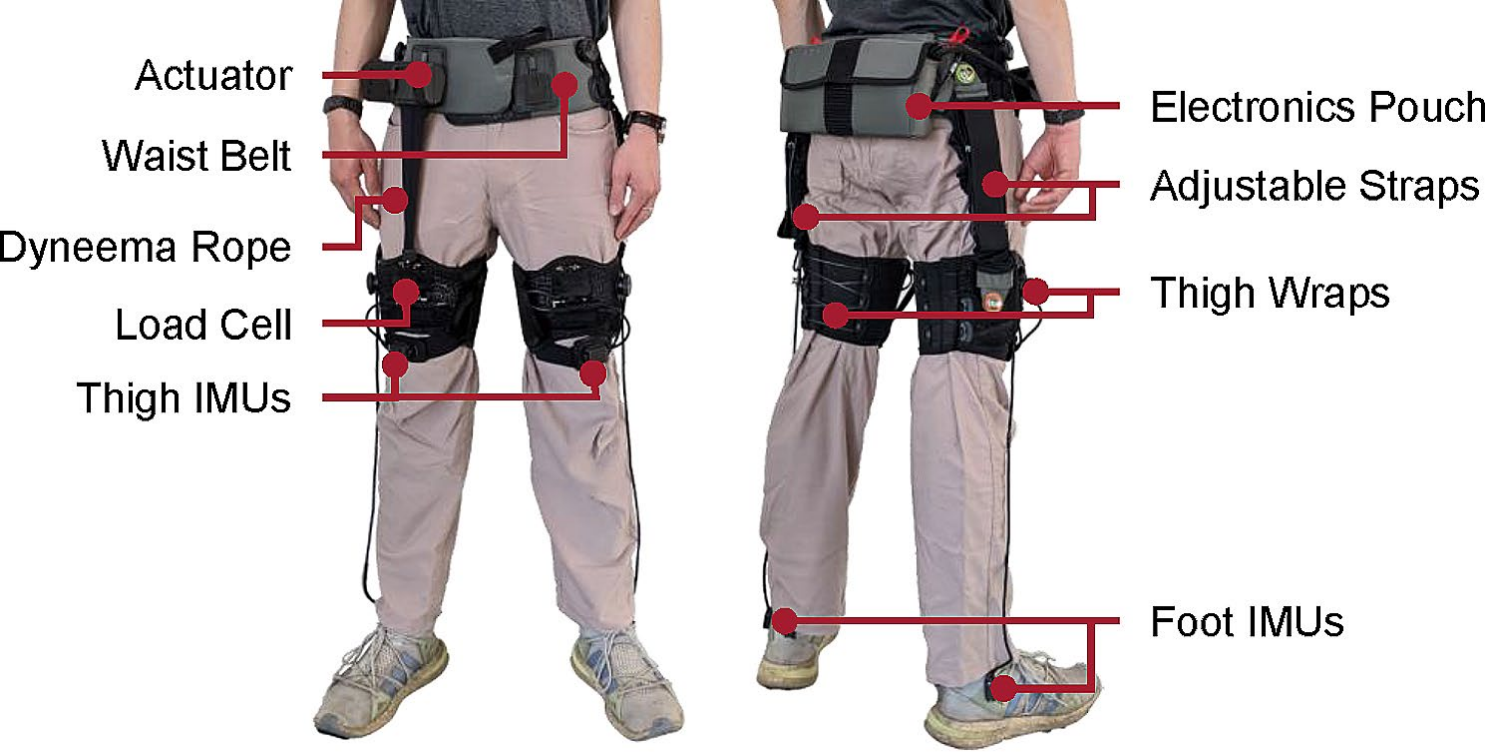
Components of the unilateral hip flexion exosuit, set up for an individual with right hemiparesis. For use in individuals with left hemiparesis, the actuator and the rope would be attached to the left side

The actuator delivered hip flexion assistance through winching a Dyneema rope spanning in front of the paretic thigh (Fig. 1). The Dyneema rope (P/N KL0200, Marlow, USA; 1.8 mm diameter) was driven by a motor (U5, T-MOTOR, China) on one end and attached to the thigh wrap on the other end via a load cell (LSB200, FUTEK, USA) and a fabric loop. The actuator generated up to 150 N tensional force, yielding a flexion moment around the hip joint.

### Exosuit controller design

**Fig. 2:**
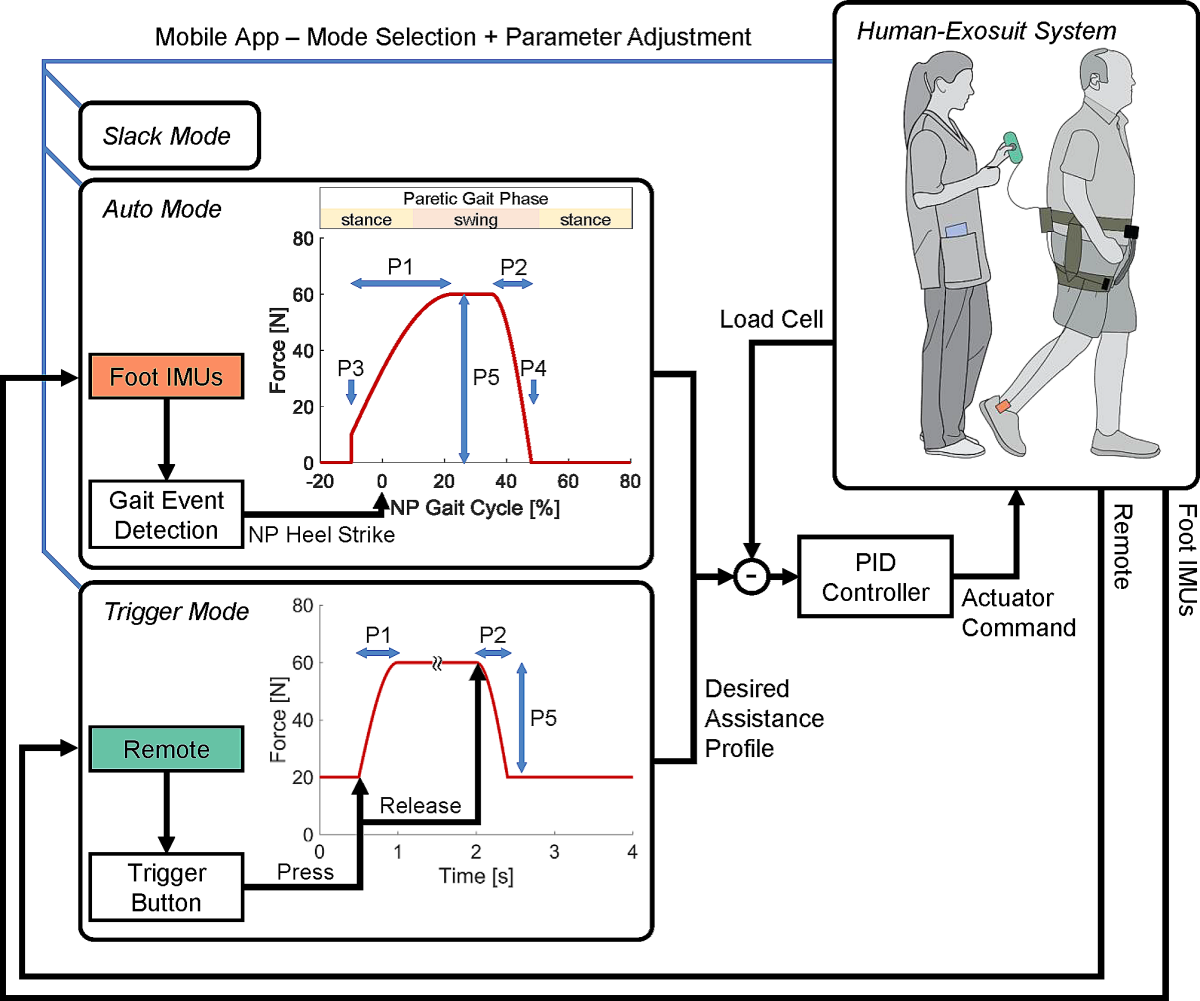
Overview of the exosuit controller. The PTs used a mobile app to select the operation modes (slack, auto, or trigger) that defined the desired assistance profile. The low-level (proportional-integral-derivative; PID) controller calculated the actuator command based on the desired assistance profile and force measured from the load cell. The mobile app also enabled the PTs to adjust assistance profile parameters, including ramp-up speed (P1), ramp-down speed (P2), onset timing (P3), offset timing (P4), and peak force magnitude (P5). NP: Non-Paretic

The controllers operated with a microprocessor (ATSAME70N21, Atmel Co, USA) mounted on the main electronic board. The exosuit delivered the hip flexion assistance with high-level and low-level controllers when it was in active condition (Fig. 2). The high-level controller determined the assistance profile in two active modes – a gait-event-based auto mode or a PT-based trigger mode.

In auto mode, the controller detected non-paretic heel strikes using the inertial measurement units (IMUs; MTi-3 AHRS, Xsens, Netherlands) mounted on both feet [52]. The foot IMUs rather than the thigh IMUs were used as the exosuit assistance force created movement of the thigh piece. We estimated the gait cycle based on previous stride times starting from the second stride and up to the most recent three strides [52] to maximize the stability of the controller [50]. The default assistance profile, which was based on a previous study [50], started in late stance and ended in late swing of the paretic limb. During early- to mid- stance when no assistance profile was prescribed, a position controller was used to keep the rope slack (approximate zero force) and trace to a pretension force (10 N) before the onset of assistance.

Since auto mode relies on gait event detection designed for consistent and predictable gait patterns, we implemented a trigger mode to accommodate severe impairment including slow and irregular gait patterns often observed in individuals during inpatient rehabilitation. With this mode, the PTs prompted the onset and offset of the assistance profile by pressing and releasing the trigger button on a remote. When not prompted, the exosuit maintained a constant baseline force (20 N) to ensure pretension, so that when a command was given by PT, there would not be slack or excess compliance in the actuator-to-human interface. The remote was connected to the main electronic board with a 1.2 m long wire and could be removed when unused.

In both modes, the low-level controller tracked the assistance profile with a proportional-integral-derivative (PID) controller and sent the actuator command to the motor driver (Gold Twitter, Elmo Motion Control Ltd, Israel) mounted on the main electronic board. The exosuit could also operate in slack mode, during which the exosuit was worn and turned on to collect sensor data but did not aid or impede participants’ motion.

### Mobile application and operation notes

For the PTs to operate the exosuit without in-person technical support during sessions, we developed a custom mobile application (app) that connected the exosuit with Bluetooth. The mobile app guided the PTs through the setup of the exosuit and displayed error messages when debugging was needed. The PTs could select operation modes and adjust assistance profile parameters based on their observation and patients’ preferences. More details about the available selections are described in additional file 3. We documented all operation notes related to aspects of fitting the exosuit, donning/doffing, setting up hardware, updating software, and troubleshooting so that the PTs could facilitate and maintain the exosuit in clinics.

### Study design

The study was carried out by registered PTs at Shirley Ryan AbilityLab (SRAlab, Chicago, IL, USA) and was approved by the Institutional Review Board of Northwestern University. All methods were carried out in accordance with the approved study protocol #IRB00210500.

The participants were recruited between August 2021 and March 2022 and were enrolled 14 to 93 days following the onset of stroke while receiving inpatient rehabilitation at the SRAlab. The standard treatment during inpatient rehabilitation included three hours of daily therapy with at least five 1-hour conventional gait training sessions per week. Recruited participants received several 1-hour walking sessions with the exosuit in addition to their standard care. The exosuit walking sessions were scheduled from the participant’s consent until the participant’s discharge or decision to opt out of the study. The discharge of the participants was determined based on the SRAlab’s regular guidelines and was independent from this study.

During all walking sessions, the PTs used handling techniques to provide balance support and limb advancement assistance as needed to ensure the safety and support ambulation of participants. For analysis, we grouped the sessions completed by all participants according to the level of assistance needed from the PTs during exosuit slack condition: Limb-Balance-support (**LB-support**) sessions during which the PTs provided both limb advancement assistance and balance support; Balance-support (**B-support**) sessions during which the PTs provided balance support only; **No-support** sessions during which the participants could ambulate independently without any support from the PTs.

At the beginning of each exosuit walking session, the participants completed the 2-minute walk test (2MWT) and the 10-meter walk test (10MWT) in exosuit slack and active conditions to obtain walking distance and speed, respectively. In the active condition, the PTs were instructed to attempt the auto mode and switch to the trigger mode if the gait detection failed. In addition to functional tests with the exosuit, the PTs also evaluated the participants’ hip flexor strength in the first and last session using a regular manual muscle test [53], with a strength of zero representing the weakest and five representing the strongest.

After the 2MWT and 10MWT in each exosuit walking session, the participants practiced overground walking, treadmill walking, and/or stair climbing with the exosuit active based on the PTs’ discretion. We did not constrain the practice content to minimize the alteration to existing gait retraining sessions, as the study was designed to determine the feasibility of implementing the exosuit in the actual practice of clinics. The PTs were able to adjust assistance profile parameters as they saw appropriate during walking sessions. This practice did not occur on the first and last day of each participant’s walking session due to time constraints.

### Study analysis

**Fig. 3:**
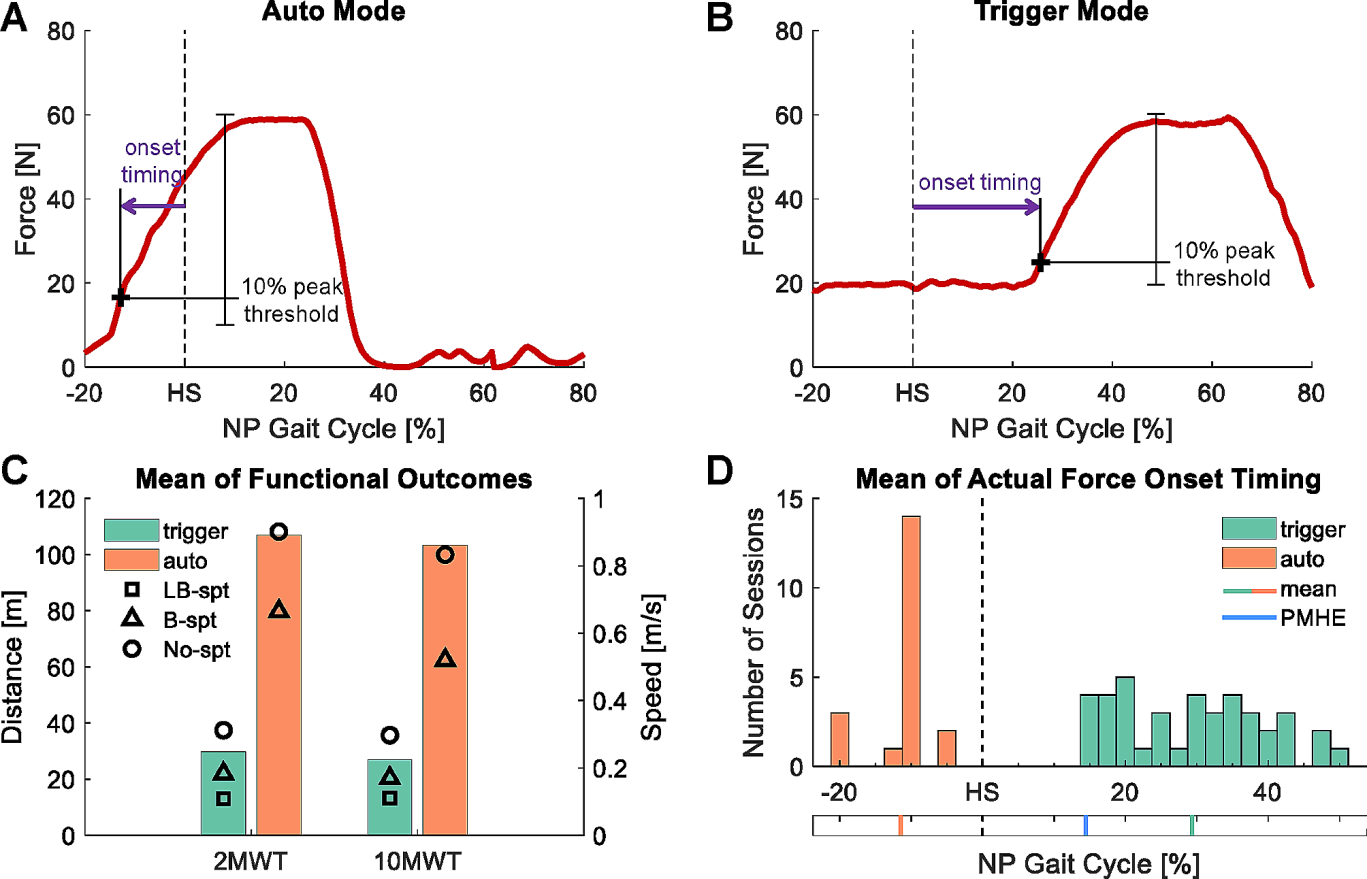
Representative force profiles for (**A**) auto mode and (**B**) trigger mode of one stride from two separate individuals/sessions. For each active mode, actual force onset timing (purple arrow) was quantified as the percentage in the gait cycle (0% at non-paretic (NP) heel strike (HS)) when the assistance force measured by the load cell exceeded the corresponding thresholds. The thresholds were set as 10% of the peak magnitude (60 N for this representative illustration) relative to the baseline or pretension force, which was set to 10 N for auto mode and 20 N for trigger mode. (**C**) We compared the functional outcomes, i.e., distance traveled during the 2MWT and walking speed during the 10MWT in exosuit active condition, between sessions using trigger mode and auto mode. Each bar represented the mean of all sessions using each active mode. We also plotted the mean of functional outcomes of each grouped session (LB-support sessions (square), B-support sessions (triangle), and No-support sessions (circle)) when using each active mode. (**D**) We compared the mean of the actual force onset timing for the auto mode (orange, 21 sessions) and trigger mode (green, 49 sessions) during the 2MWT. Each bar represented the number of sessions during which the mean of actual force onset timing fell within the bin (2.5% increment). The vertical lines at the bottom represented the mean of the sessions using each active mode, along with the mean of gait percentage at paretic maximum hip flexion (PMHE) across all sessions (blue). NP: Non-Paretic, HS: Heel Strike, spt: support, PMHE: Paretic Maximum Hip Extension

We compared the mean of actual force onset timing of assistance force during the 2MWT between the two active modes. Actual force onset timing was defined as the percentage in the gait cycle when the assistance force measured by the load cell passed 10% of the peak magnitude relative to the pretension or baseline force, equivalent to 10 N for auto mode and 20 N for trigger mode (representative profiles for each mode in Fig. 3A and B). We removed the strides with incomplete assistance profiles. Given the unequal sample size and non-normal distribution of active mode timing, we compared the mean of actual force onset timing between the two modes using the Mann-Whitney U test.

In addition to functional outcomes during the 2MWT and 10MWT, we developed a post-processing algorithm to estimate gait metrics to evaluate the spatiotemporal biomechanics of participants. The estimation algorithm was based on bilateral thigh and foot IMUs, which collected three-dimensional angle, angular velocity, and linear acceleration. By extending previous research on estimating gait events and metrics for individuals with chronic stroke walking as slow as 0.3 m/s using foot IMUs [52, 54], we incorporated frequency analysis and thigh IMUs to account for slower and less regular gait expected from individuals with subacute stroke. The estimated gait metrics included cadence (steps/min), stride length (m), and swing time symmetry (unitless). Swing time symmetry ($S{W}_{symm}$) was defined as the following:

$$S{W}_{symm}=\frac{\text{max}\left({T}_{SW,r}, {T}_{SW,l}\right)}{\text{min}\left({T}_{SW,r},{T}_{SW,l}\right)}$$

where ${T}_{SW.r}$ was the average swing time of the right side and ${T}_{SW,l}$ was the average swing time of the left side. A swing time symmetry of one indicated a completely symmetric gait and greater positive number indicated greater asymmetry [55]. We validated the accuracy of the algorithm by comparing the estimation results to GAITRite (Platinum Plus Classic, CIR Systems, USA). Details of the estimation algorithm and validation were described in additional file 4.

We evaluated the orthotic effect of the exosuit by comparing functional and spatiotemporal outcomes between exosuit slack and active conditions. This was done using a linear mixed model with exosuit condition as a fixed factor and participant as a random factor, with each outcome and each grouped session (LB-support, B-support, and No-support) as an independent model. Gait metrics were evaluated during the 2MWT, as steps taken during the 10MWT were too sparse to extract meaningful quality.

Statistical analyses were performed using SPSS (version 29, IBM Corp, Chicago, IL) with alpha = 0.05. Given the small sample size, we also reported a hedge’s g effect size as a measure of the proportion of the group who attained benefits from the exosuit assistance [56] including analyses without statistical model convergence. Effect sizes were considered small (g ≥ 0.2), medium (g ≥ 0.5), or large (g ≥ 0.8) [57].

## Results

### Participant demographics

Fourteen participants (P1-P14, eight males) with an average age of (mean ± standard deviation) 56.7 ± 11.3 years old and an average of 26.8 ± 20.2 days post-stroke consented and participated in this study (Table 1). The participants received on average 5.1 ± 1.3 exosuit walking sessions in the study. The average duration between the first to last exosuit walking session was 12.1 ± 3.5 days. Among all sessions, 13 sessions required LB-support, 20 sessions required B-support, and 39 sessions required No-support. All participants either maintained or decreased the required assistance from the PTs with progressing walking sessions. The average hip flexor strength was 0.7 ± 1.2 in LB-support sessions, 2.8 ± 1.5 in B-support sessions, and 3.6 ± 1.2 in No-support sessions.

**Table 1:** Participants and sessions demographics

	Days since stroke at the first session	Total duration of study participation (days)	Total sessions	Sessions in each group			Paretic Hip Flexor Strength	
				LB	B	No	first	last
P1	43	13	6	0	4	2	3	3
P2	39	14	5	3	2	0	0	1
P3	29	7	3	0	3	0	4	NA^*^
P4	28	16	6	3	3	0	0	2
P5	26	7	3	0	2	1	4	5
P6	27	12	7	1	0	6	3	4
P7	24	13	7	0	0	7	4	5
P8	97	13	6	0	2	4	3	4
P9	38	15	5	2	0	3	0	3
P10	38	8	4	2	2	0	0	0
P11	23	15	5	2	0	3	1	2
P12	45	6	4	0	1	3	4	5
P13	19	15	6	0	1	5	4	4
P14	27	15	5	0	0	5	2	2

^*^ P3 discharged early and did not complete additional assessment at the last exosuit walking session

### Exosuit usage

We delivered a training session with the PTs and a practice session with one individual post-stroke. After the training, the PTs were able to successfully operate the exosuit to provide hip flexion assistance in 97% (70 out of 72) of the total attempted sessions with two sessions having technical challenges. No falls or injuries were reported from using the exosuit throughout the study. The exosuit did not require in-person maintenance during the study.

Out of 70 walking sessions, 49 sessions (70%) were completed with trigger mode. For both modes, the PTs were able to identify comfortable and effective assistance profile parameters for all but one session for one participant. In general, trigger mode was used with participants with lower walking speed (on average 0.23 ± 0.12 m/s during the 10MWT in exosuit active condition, range 0.05–0.56 m/s; Fig. 3C). Participants who walked at higher speed (on average 0.86 ± 0.30 m/s during the 10MWT in exosuit active condition, range 0.29–1.51 m/s) used the exosuit with auto mode. The PTs also operated the exosuit in trigger mode during stair training in 11 sessions (two B-support, nine No-support sessions.)

### Assistance Profile of the two active modes

The mean of actual force onset timing of auto mode was − 11.4 ± 3.9%. The mean of the actual onset timing of trigger mode was 29.4 ± 10.3%, which was significantly different from that of auto mode (*p* < 0.001, g = 4.47; Fig. 3D).

### Orthotic Effect of the Exosuit

The 2MWT and 10MWT with exosuit slack were evaluated in all 72 sessions. The 2MWT in three sessions and the 10MWT in two sessions with exosuit active were not evaluated due to technical challenges and participant fatigue. Gait metrics were not available in 11 sessions with exosuit slack and 10 sessions with exosuit active due to interruption in Bluetooth communication and dropped sensors (Table 2).

**Table 2:** Number of evaluated sessions for each outcome and exosuit condition

Exosuit Condition	2MWT	10MWT	Gait Metrics (2MWT)
Slack	72	72	61
Active	69	70	62

### Functional outcomes

**Fig. 4:**
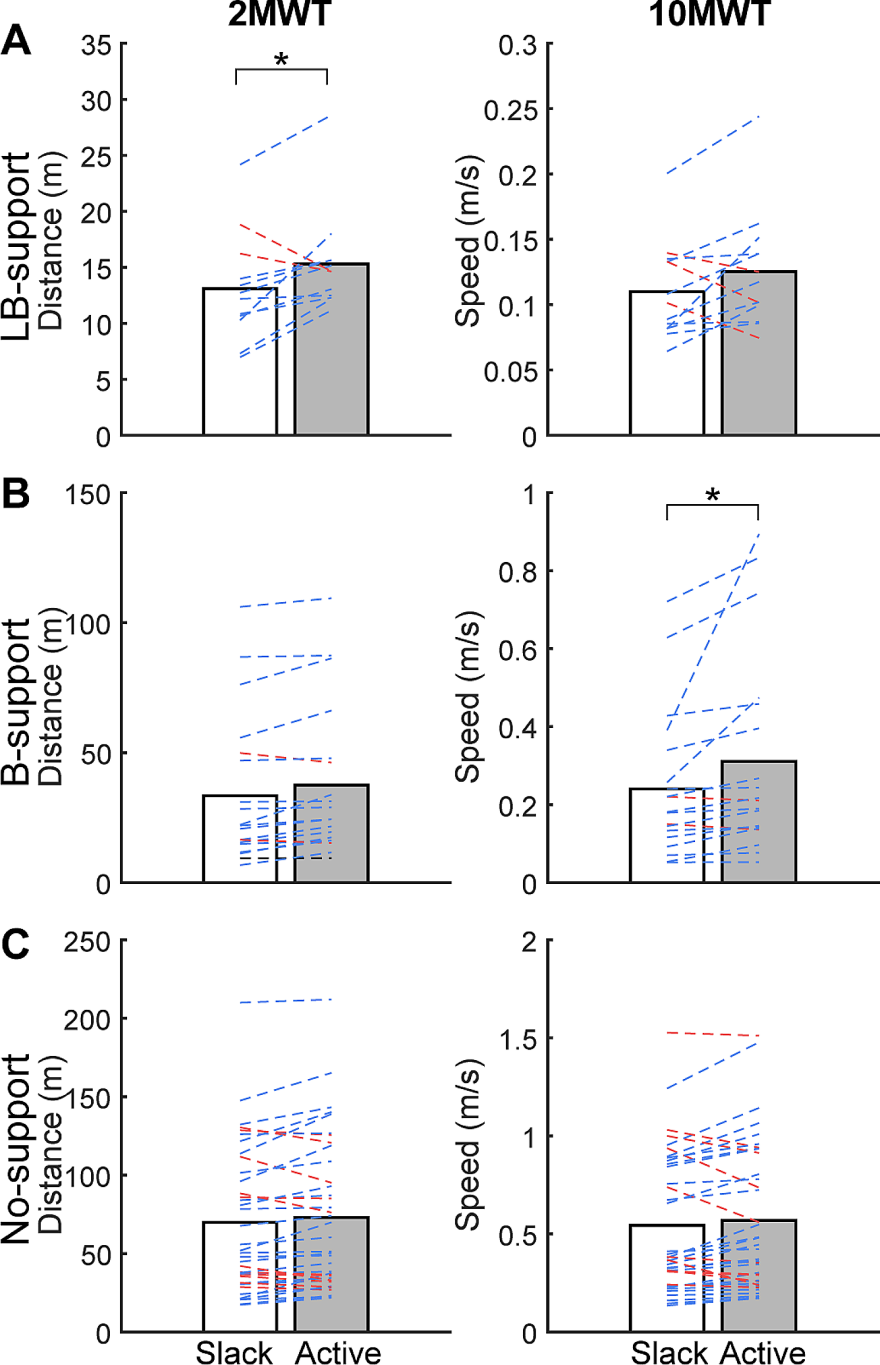
Average outcomes of the 2MWT and the 10MWT for (**A**) LB-support, (**B**) B-support, and (**C**) No-support across all sessions in exosuit slack (white) and exosuit active (gray) conditions. Individual sessions are represented with blue (positive orthotic effect), red (negative orthotic effect), or black (neutral orthotic effect) lines. Significant exosuit orthotic effects in the group average are noted with * (*p* < 0.05)

During all LB-support sessions, with the exosuit active the participants were able to advance their limb and ambulate without manual limb advancement assistance from the PTs. With the exosuit slack, during which the PTs provided both manual limb advancement and balance support, the participants completed the 2MWT with an average distance of 13.1 ± 4.6 m and the 10MWT with an average speed of 0.11 ± 0.04 m/s. With the exosuit active, during which the PTs provided balance support only, the average distance traveled during the 2MWT significantly increased by 2.2 m ± 3.1 m compared to exosuit slack (*p* = 0.017, g = 0.47; Fig. 4A). However, the average speeds during the 10MWT were similar between the two exosuit conditions (*p* = 0.062, g = 0.34).

In B-support sessions, during which the PTs provided balance support in both exosuit conditions, the participants completed the 2MWT with an average distance of 33.4 ± 28.1 m and the 10MWT with an average speed of 0.24 ± 0.18 m/s with exosuit slack. Average speed during the 10MWT significantly increased by 0.07 ± 0.12 m/s with exosuit active compared to exosuit slack (*p* = 0.042, g = 0.27; Fig. 4B). However, the average distances during 2MWT were similar between the two exosuit conditions (*p* = 0.173, g = 0.14).

In No-support sessions, the participants completed the 2MWT with an average distance of 69.9 ± 45.6 m and the 10MWT with an average speed of 0.54 ± 0.35 m/s with exosuit slack. Both the average distances during the 2MWT and the average speeds during the 10MWT were similar between the two exosuit conditions (*p* > 0.473, g = 0.07; Fig. 4C).

### Spatiotemporal gait metrics during 2MWT

**Fig. 5:**
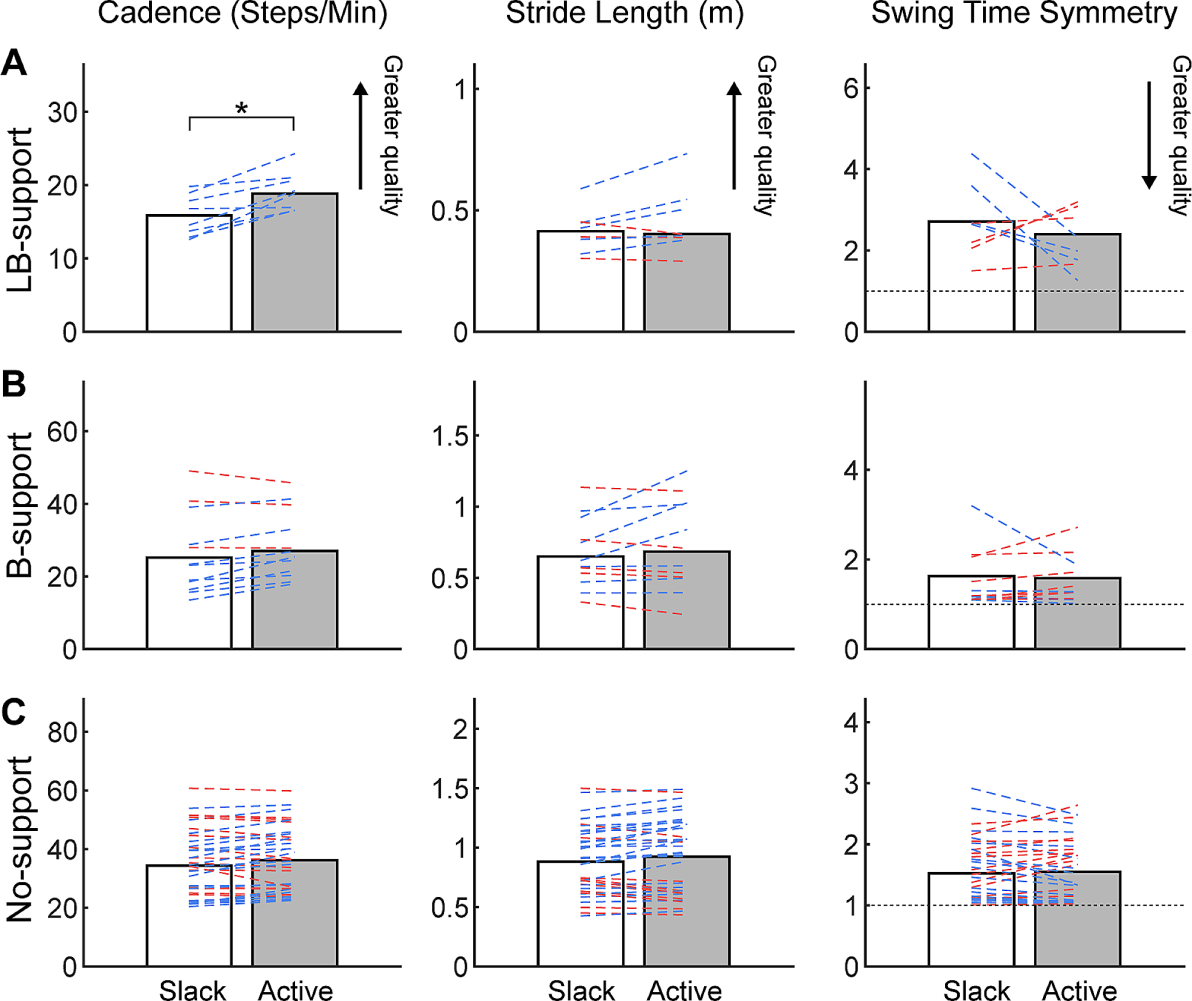
Average estimated gait metrics obtained from exosuit IMU during 2MWT for (**A**) LB-support, (**B**) B-support, and (**C**) No-support across all sessions in exosuit slack (white) and exosuit active (gray) conditions. Individual sessions are represented with blue (positive orthotic effect, i.e., increased cadence and stride length, decreased (closer to 1) swing time symmetry), red (negative orthotic effect), or black (neutral orthotic effect) lines. Significant exosuit orthotic effects in the group average are noted with * (*p* < 0.05)

In LB-support sessions, the participants completed the 2MWT with an average cadence of 15.9 ± 2.8 steps/min, stride length of 0.41 ± 0.09 m, and swing time symmetry of 2.7 ± 0.9 with exosuit slack. Their cadence significantly increased by 3.4 ± 2.1 steps/min with exosuit active compared to exosuit slack (*p* = 0.003, g = 0.94; Fig. 5A), while stride length (*p* = 0.121, g = 0.09) and swing time symmetry (g = 0.36) were similar between the two exosuit conditions.

In B-support sessions, the participants completed the 2MWT with an average cadence of 25.2 ± 10.9 steps/min, stride length of 0.65 ± 0.24 m, and swing time symmetry of 1.6 ± 0.9 with exosuit slack. In No-support sessions, the participants completed the 2MWT with an average cadence of 34.4 ± 11.7 steps/min, stride length of 0.88 ± 0.29 m, and swing time symmetry of 1.5 ± 0.5 with exosuit slack. These gait metrics were similar between the two exosuit conditions in both B-support (*p* > 0.066, g < 0.17; Fig. 5B) and No-support (*p* > 0.339, g < 0.16; Fig. 5C) sessions.

## Discussion

In this study, we demonstrated that a unilateral exosuit for hip flexion assistance can be implemented in clinics or hospitals to aid paretic limb advancement during inpatient gait retraining for individuals post-stroke. However, the impact on walking speed and distance when wearing the device was variable across participants. PTs could operate the exosuit without in-person technical support and use it to assist limb advancement over a diverse range of activities in patients with different levels of impairment. Using this exosuit eliminated the need for manual limb advancement in walking sessions where participants could not ambulate without such assistance from the PTs. Furthermore, we observed several orthotic benefits in walking sessions where participants required PT support to ambulate.

We designed the textile and hardware of the exosuit to maximize the clinical usability by PTs. The PTs were able to don our exosuit within two minutes and expressed confidence with its operation within a single training session. Although existing gait assistance or training devices provide greater levels of support, they have a donning time of as long as 30 min and are reported to have a high learning curve [37–39]. Since the allotted time for gait retraining sessions is finite, excessive donning times impact the time patients can dedicate to receiving mass practice. A high learning curve, which was reported from both the PTs’ and the patients’ perspectives [37–39], may discourage clinical implementation of assistive devices. Furthermore, we implemented a trigger mode, along with auto mode, that allowed the PTs to manually control the onset and offset of exosuit assistance. We had anticipated that trigger mode would be particularly important for inpatient rehabilitation, as electromechanical devices that neither support weight nor enforce motion often face challenges detecting and assisting severely impaired gait [58–61]. As expected, average walking speed and distance during functional outcomes were lower in sessions that used trigger mode compared to those that used auto mode. More importantly, trigger mode was used in 70% of the walking sessions, suggesting that the use of trigger mode enabled individuals with severe impairment to use the exosuit who otherwise could not use similar assistive devices. The PTs also used trigger mode for gait retraining on stairs, during which auto mode would require an additional activity detection algorithm. This highlights the potential to use the exosuit during advanced activities to maintain intensity in gait retraining as individuals post-stroke recover, which could maximize rehabilitation outcomes [62, 63]. That said, the usability of the exosuit may be further improved in the future with a controller that can automatically provide assistance for patients with a wide range of mobility and during different activities to reduce the cognitive load on PTs associated with the trigger mode.

The exosuit successfully eliminated the need for manual limb advancement during walking sessions where participants required such assistance from the PTs to ambulate (LB-support). The exosuit assistance was sufficient to support individuals to independently advance their limbs, and thus may have eliminated awkward posture and repetitive motion that are typically associated with manual limb advancement provided by the PTs [11, 16, 64]. A previous study has demonstrated that an assistive device designed to counteract gravity during upper extremity training reduced muscular activity and cardiac effort from the PTs [65]. Although we did not explicitly quantify the effect of our exosuit on the fatigue experienced by the PTs, we may expect a similar reduction in their physical effort as the exosuit assistance essentially substituted manual limb advancement assistance. Future studies are needed to evaluate the benefits of using exosuits during inpatient rehabilitation for both the PTs and the patients through surveys inquiring about device usability, engagement, and burden.

The elimination of PT manual assistance during LB-support sessions also likely positively influenced rehabilitation outcomes for the patients. The fatigue experienced by the PTs [18, 35] and the variability inherent to manual assistance [66] can limit the effectiveness and quantity of mass practice. Likely in part attributed to the elimination of this manual assistance, we observed improvements in distance and cadence during the 2MWT in LB-support sessions. While statistically significant, it is worth noting that the orthotic effect in distance is less than the minimal clinically important difference (MCID) for the 2MWT. This value was established from individuals in later stages of stroke recovery with a greater average gait capacity of 130 m traveled during the 2MWT [67]. Given the substantially lower mobility (average distance traveled of 13 m) for individuals in LB-support sessions, we believe the established MCID may not be an appropriate point of reference. Future work should evaluate the clinically relevant value of exosuits to quantify the ability to promote greater endurance and quantity of mass practice in an inpatient setting.

Using the exosuit provided an orthotic benefit on walking speed with exosuit assistance in a subgroup of walking sessions explored in this study (B-support). The participants in B-support sessions required balance support from the PTs and exhibited an average of “fair (grade 2.8 out of 5)” hip flexor strength [53]. Since weakened hip flexor strength is negatively associated with walking speed [68], we may interpret that the exosuit assistance supplemented the hip flexor strength of the participants during B-support sessions. Although the participants in LB-support sessions also exhibited weakened hip flexors (an average of “trace (grade 0.7 out of 5)”), there was no significant orthotic effect on walking speed in these sessions. This might be explained by the fact that in LB-support sessions, participants received assistance in both exosuit slack and active conditions – slack condition with manual assistance from the PTs, and active condition with assistance from the exosuit. Therefore, the lack of orthotic effect suggests the exosuit assistance was comparable to assistance manually provided by the PTs. In contrast, the participants in No-support sessions, who had an average of “good (grade 3.6 out of 5)” hip flexor strength, did not improve their walking speed with the exosuit as a group, suggesting that the additional hip flexion assistance was not beneficial for individuals with sufficient limb advancement capacity. Similarly, prior work with exoskeletons providing hip assistance without body weight support demonstrated its ability to improve walking speed in some, but not all individuals with chronic stroke [69, 70]. Our results suggest that the positive effect of joint-targeting exosuit assistance demonstrated in individuals in later stages of stroke recovery may also be anticipated in individuals in earlier stages of recovery. This is particularly exciting, as a previous study demonstrated that gait retraining with increased walking speed enforced by assistance could result in a better outcome in chronic stroke recovery [71]. Therefore, the use of hip-assisting exosuits may as well help those in the early stages of recovery obtain better outcomes during inpatient rehabilitation and further influence their reintegration into the community following discharge.

Besides the improved distance and cadence in LB-support sessions and walking speed in B-support sessions, however, the majority of the functional and spatiotemporal outcomes were similar with and without exosuit assistance across all sessions. The positive orthotic effects exhibited in LB- and B-support sessions but not in No-support sessions were consistent with previous studies where individuals with higher functional scores [43, 46, 72] or in later stages of stroke recovery [34, 73, 74] benefited less from assistive devices. That said, the exosuit assistance did not disturb or negatively impact gait performance in individuals who did not necessarily require limb advancement assistance. Moreover, it is important to note that the exosuit walking sessions in our study were analyzed as three groups based on the level of assistance the participants required from the PTs. While there were no significant orthotic effects as a group, there was notable variability in the individual response of functional outcomes. Specifically, participants in 77% of LB-support sessions, 80% of B-support sessions, and 69% of No-support sessions improved their 10MWT speed or 2MWT distance with exosuit assistance. Several previous studies also reported similar trends where the responses to assistive devices were heterogeneous among individuals post-stroke [50, 69, 70]. This suggests that the amount of exosuit benefit to an individual post-stroke is likely not solely influenced by the required assistance level from the PTs, but rather dependent on several patient-specific factors. The numerous potential factors that can influence exosuit benefits calls for future investigations with a larger sample size.

One of the important factors to consider when understanding the effect of assistive devices is the force magnitude and timing of the provided assistance. In our study, there was a notable difference between the assistance provided by the two active modes. We pre-programmed the assistance onset timing for auto mode to occur before non-paretic heel strike based on our previous study with individuals with chronic stroke [50]. However, the PTs manually controlled the onset of trigger mode assistance based on each individual and each stride. Our results indicate that on average, the PTs applied the trigger 41% later in the gait cycle compared to auto mode. Furthermore, trigger mode maintained a baseline force during the entire gait cycle due to the low-level controller requirement, while auto mode remained slack during the early stance and started pretension before the onset of assistance in late stance. Although the higher baseline force with trigger mode and earlier onset of force with auto mode might have acted as resistance on the hip during stance, the PTs did not qualitatively observe restriction or disturbance. In spite of the differences in force profiles between the two active modes, we are unable to directly compare the effect of the chosen active mode on the functional or biomechanical outcomes as only one mode was used in a single session. Additionally, the comparison of the orthotic effect across sessions was difficult since the selected active mode and the assessment outcome were affected by the walking ability of participants that varied significantly across sessions. That said, we understand from previous studies that different assistance profiles could achieve various gait benefits for different populations [70, 75]. The diversity of assistance profiles explored in different populations motivates further investigation of approaches to optimizing hip flexion assistance according to targeted gait kinematics and outcomes.

There are several limitations to consider when interpreting the results of this study. First, we quantified the biomechanical outcomes in a relatively small cohort using only a subset of estimated metrics that could be easily acquired in the clinic and properly validated. The smaller effect sizes observed for many conditions that lacked statistical significance warrant additional studies focused on more specific subgroups of this population and in more controlled conditions. Moreover, it is possible that other biomechanical measures, such as step length, step width, trailing limb angle, and propulsion [76, 77], are also important to characterize post-stroke gait and may help interpret the exosuit effect. However, these metrics are difficult to acquire without laboratory equipment or estimate from body-worn sensors. Second, there was only minimal individualization of the assistance profile in our study. Individual customization of assistance profiles is useful in ensuring maximal benefits from assistive devices [75, 78, 79]. We implemented the exosuit to allow certain customization as the PTs had the ability to decide the onset of offset timing in the trigger mode and tune the parameters of the assistance profiles. However, finding the optimal assistance manually was not realistically possible due to the limited time and capacity of PTs during gait retraining and the broad range of parameter space.

## Conclusions

In this work, we demonstrated the successful implementation of a unilateral hip flexion exosuit during inpatient rehabilitation for individuals post-stroke. The exosuit was easily operated by the PTs and eliminated the need for manual limb advancement assistance. While we did not see large group-level changes in functional and spatiotemporal measures, we still observed positive orthotic effects in the majority of sessions. Together, our results indicate the use of hip flexion exosuit is a promising avenue to explore as a tool to promote gait retraining during inpatient rehabilitation post-stroke. Devising an approach that enables delivery of optimal assistance based on specific patient needs would be imperative in accommodating heterogenous gait impairment in individuals post-stroke. Future studies should also investigate the long-term therapeutic effect of inpatient use of this exosuit as well as explore its potential application in outpatient rehabilitation and as a mobility aid in ecological settings.

### Electronic supplementary material

Below is the link to the electronic supplementary material.

Additional Video 1: Is a movie file showing the assembly process of the exosuit

Additional Video 2: Is a movie file showing the donning process of the exosuit

Additional File 3: Is a word document describing the details of the mobile application

Additional File 4: Is a word document describing the details and validation of the gait metric estimation algorithm

## Data Availability

The datasets used and/or analyzed during the current study are available from the corresponding author on reasonable request.

## Abbreviations

PT: Physical Therapist
PBWSTT: Partial Body Weight Supported Treadmill Training
IMU: Inertial Measurement Unit
PID: Proportional-Integral-Derivative
LB: Limb-Balance
B: Balance
2MWT: 2-Minute Walk Test
10MWT: 10-Meter Walk Test
NP: Non-paretic
HS: Heel Strike
PMHE: Paretic Maximum Hip Extension
