# Supplementary material for: Implementation of a unilateral hip flexion exosuit to aid paretic limb advancement during inpatient gait retraining for individuals post-stroke: a feasibility study
Source: J Neuroeng Rehabil. 2024 Jul 18;21:121. doi: 10.1186/s12984-024-01410-0 (PMC11256417; doi:10.1186/s12984-024-01410-0)
Supplement: Supplementary file 3 — Additional File 3: Is a word document describing the details of the mobile application [file 12984_2024_1410_MOESM3_ESM.docx]

# Additional File 3. Mobile Application

The mobile application (app) used by the PTs in this study was developed in the Android operating system. The app was connected to the exosuit via Bluetooth. With the app, the PTs selected the paretic side (left/right) and the operation mode (slack/auto/trigger - displayed as “manual”; Figure S1A). Given the operation mode, the PTs could adjust the assistance profile parameters in the app.

For the convenience of the PTs, the selections of peak force magnitude and preset combinations of timing parameters (default/fast) were shown on the same page of mode selection. The PTs could further adjust each parameter in a separate page visualizing the assistance profile (Figure S1B, Table S1).


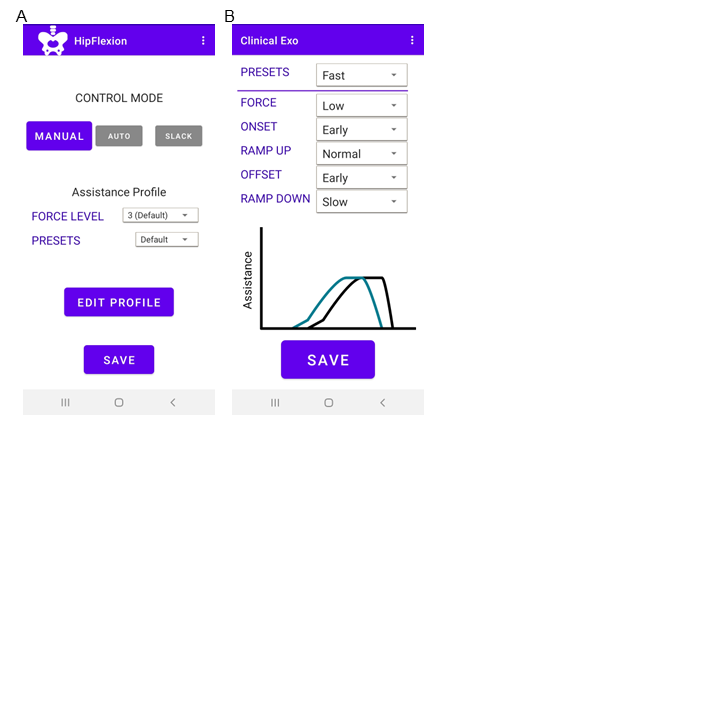


**Figure S1**. The screenshots of the mobile app for (A) mode selection (manual, auto, or slack), preset assistance profile selection (default or fast), and force level selection, and (B) parameter adjustment with the visualization of the current (black) and selected (blue) assistance profile.

**Table S1**. Options for profile parameters. The preset combinations of timing parameters were highlighted with bold for “default” and underlined for “fast”.

|  | Auto | Trigger |
| --- | --- | --- |
| Peak force magnitude | Level 1 to 7 (default **3**) | Level 1 to 7 (default **3**) |
| Ramp-up speed | Fast, **Normal**, Slow | Fast, **Normal**, Slow |
| Ramp-down speed | Fast, **Normal**, Slow | Fast, **Normal**, Slow |
| Onset timing | Early, **Normal**, Late | NA |
| Offset timing | Early, **Normal**, Late | NA |
