# Supplementary material for: Implementation of a unilateral hip flexion exosuit to aid paretic limb advancement during inpatient gait retraining for individuals post-stroke: a feasibility study
Source: J Neuroeng Rehabil. 2024 Jul 18;21:121. doi: 10.1186/s12984-024-01410-0 (PMC11256417; doi:10.1186/s12984-024-01410-0)
Supplement: Supplementary file 4 — Additional File 4: Is a word document describing the details and validation of the gait metric estimation algorithm [file 12984_2024_1410_MOESM4_ESM.docx]

# Additional File 4. Gait Metric Estimation

We developed a custom post-processing algorithm in MATLAB (2021b, Mathworks, USA) to estimate gait quality metrics, including cadence (steps/min), stride length (m), and swing time symmetry (unitless). Cadence and stride length estimation were based on gait segmentation using the non-paretic side.

## Estimation of cadence and swing time symmetry

We identified foot contact and toe off events by identifying the peaks of the foot gyroscope in the sagittal plane (1). To ensure the identified peaks correspond to the gait events, we set the minimum peak distance based on gait frequency obtained from Fourier transform of the foot and thigh segmental angles in the sagittal plane. We further filtered the detected gait events so that each stride only contained a single foot contact and toe off from both legs. Cadence was calculated as the inverse of the time between adjacent toe off events and swing time was calculated as the time between adjacent toe off and heel strike events.

## Estimation of Stride Length

We obtained the position of the foot by integrating the foot segmental acceleration obtained from the IMUs and applying a zero velocity update mid-stance (2). The thresholds used in the zero velocity update for analyzing our dataset were larger than what were typically reported in previous work (2) due to increased variability during mid-stance in our participants. The stride length was calculated as the horizontal distance between the position of the foot at adjacent contralateral toe off.

## Validating accuracy of estimation

We evaluated the accuracy of the gait metric estimation by comparing the estimated metrics to data obtained from GAITRite. GAITRite (Platinum Plus Classic, CIR Systems, USA) was a sensorized walking mat of 4.27m used to evaluate gait metrics including cadence, stride length, and swing time. GAITRite data was only collected in sessions during which participants could advance their limb independently (B- or No-support) on each participant’s first and last exosuit walking sessions - 13 sessions from seven participants. The average walking speed of these sessions was 0.74 ± 0.47 m/s (range 0.15 - 2.34 m/s).

Considering data obtained from GAITRite as ground truth, we quantified the accuracy of our gait metric estimation by determining the correlation between estimated metric and ground truth using linear regression. While each data point represented an average metric at each walking bout, the relative contribution of these data points for linear regression was weighted based on the number of strides in each bout. Pearson’s correlation coefficient was considered “strong” (r > 0.7), “moderate” (0.3 < r < 0.7), or “weak” (r < 0.3) with alpha = 0.05 (3). We also inspected the correlation between the estimation error and walking speed to determine whether estimation was valid across a breadth of walking speeds. We interpreted that if the estimation error was not correlated with walking speed (i.e., the error was not greater in lower walking speed), the estimation algorithm could be applied to individuals with lower mobility.

Based on the strong positive correlation (R > 0.95, p < 0.01) between estimated metrics and ground truth obtained from GAITRite (Figure S2A) as well as the lack of correlation between estimation error and walking speed (Figure S2B), we interpreted that the estimated metrics may be appropriately used to determine gait quality during walking sessions with the exosuit. Of note, stride length error was negatively correlated to the walking speed (R^2^ = 0.58, p < 0.01) such that walking sessions with slower walking speed had smaller estimation error.


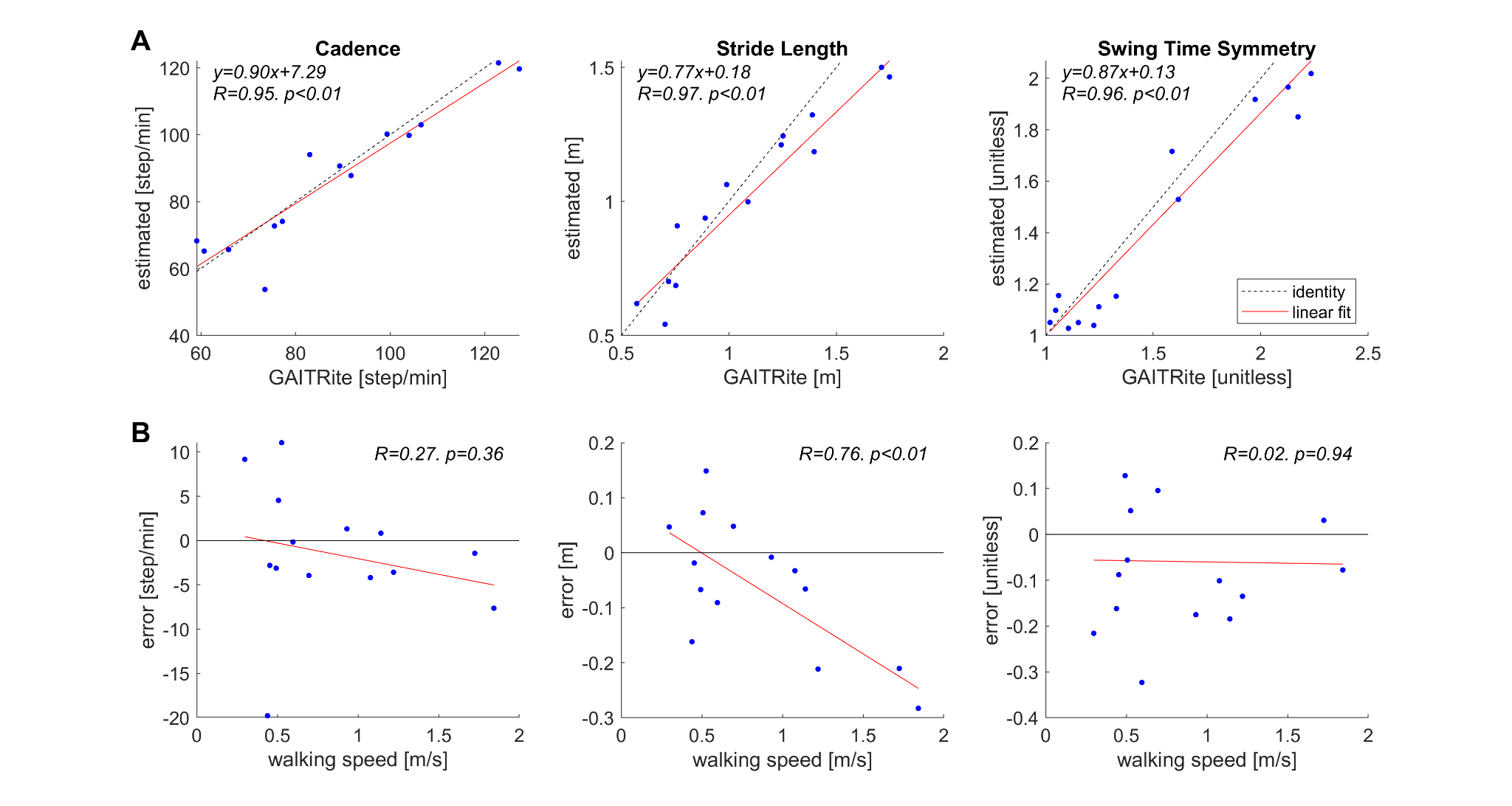


**Figure S2**. (A) Correlation between gait metrics obtained from GAITRite and estimated from IMUs. (B) The correlations between the estimation error and walking speed.

## References

1. Bae J, Siviy C, Rouleau M, Menard N, Odonnell K, Geliana I, et al. A Lightweight and Efficient Portable Soft Exosuit for Paretic Ankle Assistance in Walking After Stroke. In: 2018 IEEE International Conference on Robotics and Automation (ICRA). IEEE; 2018. p. 2820–7.

2. Arens P, Siviy C, Bae J, Choe DK, Karavas N, Baker T, et al. Real-time gait metric estimation for everyday gait training with wearable devices in people poststroke. Wearable Technol. 2021;2:e2.

3. Akoglu H. User’s guide to correlation coefficients. Turkish J Emerg Med. 2018;18(3):91–3.
